# Comparison of Ablation Zones among Different Tissues Using 2450-MHz Cooled-Shaft Microwave Antenna: Results in *Ex Vivo* Porcine Models

**DOI:** 10.1371/journal.pone.0071873

**Published:** 2013-08-12

**Authors:** Wenbin Zhou, Mengdi Liang, Hong Pan, Xiaoan Liu, Yanni Jiang, Yufeng Wang, Lijun Ling, Qiang Ding, Shui Wang

**Affiliations:** 1 Department of Breast Surgery, The First Affiliated Hospital with Nanjing Medical University, Nanjing, Jiangsu, China; 2 Department of Radiology, The First Affiliated Hospital with Nanjing Medical University, Nanjing, Jiangsu, China; 3 Jiangsu Yurun Meat Industry Group Co., Ltd., Nanjing, Jiangsu, China; Icahn School of Medicine at Mount Sinai, United States of America

## Abstract

**Background:**

For complete tumor ablation in different tissues, it is necessary to investigate the exact coagulation zone of microwave ablation in different tissues. The aim of this study was to compare the extent of microwave ablation zone in muscle, liver and adipose tissue in *ex vivo* porcine models and assess the shape of microwave coagulation zone among these tissues.

**Materials and Methods:**

Microwave ablations were performed in *ex vivo* porcine muscle, liver and adipose tissue using 2450-MHz cooled-shaft microwave antenna. The content of water, fat and protein in these three tissues was determined. Two power increments (40 and 80 W) and five time increments (1, 3, 5, 7, and 10 minutes) were used in this study. Diameters and shapes of the ablation zones were assessed on gross specimens.

**Results:**

The average percentages of water, fat and protein in these three tissues were significantly different (*P* < 0.001), respectively. The long-axis and short-axis diameters among these three tissues at each time-power combination were not significantly different (*P* > 0.05). The coagulation zones were all elliptical in muscle, liver and adipose tissue. When microwave ablation was performed in the tissue containing both muscle and adipose tissue, the coagulation zone was also elliptical. Regardless of the output power, the ellipticity index (EI) value of 1 minute treatment duration was higher than that of 10 minutes treatment duration (*P* < 0.05). Furthermore, the EI value did not decrease significantly when the treatment duration was more than 5 minutes (*P* > 0.05).

**Conclusion:**

The extent of microwave ablation zones was not significantly different among completely different tissues. Microwave ablations with ≥ 5 minutes time duration can induce coagulation zones with clinical desirable shape. Future clinical studies are still required to determine the role of microwave ablation in different tissues.

## Introduction

Various techniques of imaging-guided ablation, including radiofrequency ablation (RFA), microwave ablation, high-intensity focused ultrasound (HIFU), cryotherapy, and laser therapy, have been used in the treatment of different tumors [1–8]. Of these minimally invasive therapies, RFA and microwave ablation have been accepted as effective therapies for the treatment of hepatic tumors [1,2,6]. Compared with RFA, microwave ablation shows improved convection profile, larger ablation zones, shorter ablation durations [9,10]. Otherwise, microwave ablation is simple and convenient to perform, and the ablated zone is regularly elliptical [7]. Due to the advantages of microwave ablation [7,9,10], there is a growing trend to apply it in the treatment of various tumors.

Except for liver tumors, microwave ablation has been reported for the treatment of other solitary tumors in studies with a small sample size or in phase I ablation-resection studies [7,11–13]. Our previous phase I study suggests that microwave ablation of small breast cancer was feasible and safe [7]. However, the exact coagulation zone was not very clear among individuals in our previous study. Because of the complex anatomic structure of the breast, represented by an association of closely juxtaposed fat and glandular and fibrous connective tissues, breast cancers are always surrounded by tissues with heterogeneous conductivity [14]. For example, the main component of the breast in young patients is breast gland, with little adipose tissue; however, the main component is adipose tissue in old patients. Furthermore, the percentage of water in breast carcinoma is more than that in breast tissue and adipose tissue [7,15]. For complete ablation of breast cancer with a safety margin, the coagulation zone may contain breast carcinoma, breast gland, and adipose tissue. Therefore, it is important to investigate the exact coagulation zone of microwave ablation in different tissues with different contents.

To the authors’ knowledge, the effect of tissue heterogeneity on the extent of the ablation zone in microwave ablation is not very clear. A previous *ex vivo* study suggests that preinjected fluids did not benefit microwave ablation [16]. Another *ex vivo* study suggests that the extent of the ablation zone in microwave ablations was less affected by tissue heterogeneity than that in RFA only based on histological examination of the shape of the margin between the coagulation zone and the transition zone [17]. Up to now, there is no study comparing ablation zones *ex vivo* in different tissues using different power and time increments.

In the present study, the extent of microwave ablation zone in muscle, liver and adipose tissue in *ex vivo* porcine models was compared, and the shape of the microwave coagulation zone in these three different tissues was also assessed. In addition, the content of water, fat and protein in porcine muscle, liver and adipose tissue were tested respectively.

## Materials and Methods

### Experiment setting

**Figure 1:**
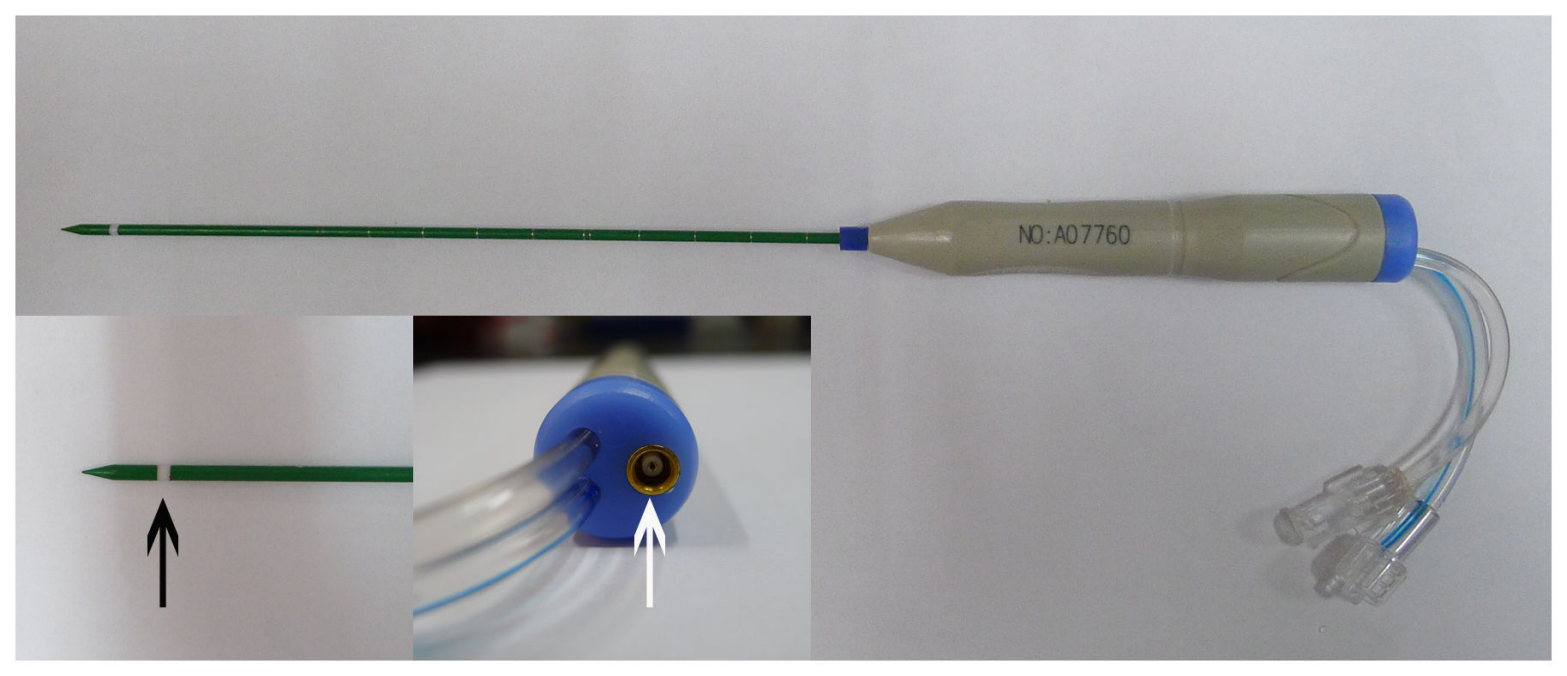
Cooled-shaft microwave antenna used in this study. The radiating segment (black arrow) embedded in the front, and the end socket (white arrow) was used to connect the plug of cable coming from the microwave generator.

Experiments were performed in *ex vivo* porcine muscle, liver and adipose tissue with a microwave applicator described previously (Nanjing Yigao Microwave Electric Institue, Nanjing, China) [7,18]. The cooled-shaft antenna, with two lumina in the shaft for circulating the cooling water, was shown in Figure 1. The irradiation frequency of this system was 2,450 MHz. 40 W was chosen according our own experience for the treatment of small breast cancer. For complete ablation, high power should be investigated. Given the relatively small necrosis obtained at 80W for 10 minutes, 80 W was also chosen in this study. The duration of energy application was varied from 1 to 10 minutes (five time points: 1, 3, 5, 7, and 10 minutes). Fresh muscle, liver and adipose tissue were obtained from a slaughter house (Jiangsu Yurun Meat Industry Group Co., Ltd.), and permission to use these animal parts was obtained from this slaughter house. The adipose tissue was from the back of the swine with a thickness of more than 4 cm. Before microwave ablation, about 30 mg of each type of tissues were cut and sent to determine the content of water, fat and protein. After each microwave ablation, the coagulation zones were dissected and sectioned along the antenna shaft. The tissue sections were incubated for cell viability in the incubation medium with 2% 2,3,5-triphenyl tetrazolium chloride (Sigma) [19]. The long-axis diameter (along the antenna) and the short-axis diameter (perpendicular to the aerial) of each ablation zone were assessed macroscopically with calipers. Finally, the sizes of coagulation zones among these three tissues were compared, and the shapes of coagulation zones were also assessed. To confirm the shape of microwave ablation in tissues with different components, additional microwave ablation was performed in the tissue containing both muscle and adipose tissue at 80 W for 3 minutes.

### Microwave ablation protocols

Under direct visualization, the microwave antenna was inserted from the side into the muscle, liver or adipose tissue at least 7 cm into the tissue to provide a satisfactory margin for even the largest ablations. Ablation overlap was avoided under ultrasonography (US) guidance with a distance larger than 5 cm according to ablation at 80 W for 10 minutes when one specimen was used for multiple ablations. Similar to previous studies [19,20], vessels larger than 5 mm were avoided by using US. Microwave ablations (n = 90) were performed in room temperature for a range of power settings and treatment durations. Two power increments (40 and 80 W) and five time increments (1, 3, 5, 7, and 10 minutes) were used, constituting 10 time-power combinations. For each combination of parameters, trials were performed in triplicate. Therefore, 30 ablations were performed in every tissue, and a total of 90 ablations were carried out. When the water cycling system well worked (the temperature of antenna below 43 °C), the microwave ablation procedure started. All microwave ablation procedures were performed by the first author with 3 years of experience in microwave ablation.

### Evaluation methods and definitions

The content of water in these three tissues was determined by direct drying method, and the content of protein was determined by the Kjeldahl nitrogen determination method. Furthermore, the percentages of fat in muscle, liver and adipose tissue were determined by acid-hydrolysis method.

After microwave ablation, the coagulation zones were dissected and sectioned along the antenna shaft. All tissue sections were incubated for cell viability in the incubation medium with 2% 2,3,5-triphenyl tetrazolium chloride. The incubation was carried out in room temperature for 30 minutes. After incubation, viable cells with mitochondrial enzyme activity were stained red, while nonviable cells were white. According to previous studies [20–24], the white zone was considered to be the ablated area. The long-axis diameter and the short-axis diameter of each ablation zone were measured macroscopically with calipers. The diameters were evaluated by two pathologists with more than 10 years of experience in pathologic examination independently. Disagreements were resolved after discussion.

The ellipticity index (EI) was calculated for all ablative zones. According to previous studies [25,26], the EI value was calculated by using long-axis and short-axis diameters with the equation EI = long-axis diameter/short-axis diameter. A spherical coagulation zone may be more suitable than a ellipsoid zone for clinical use [20]. Clinically desirable ablation shape was defined as a coagulation zone with the EI value less than 1.5 in this study.

### Statistical analysis

Numerical data were reported as means ± standard deviation (SD). One-way Analysis of Variance (ANOVA) was used to identify differences of long-axis diameter and short-axis diameter among these three different tissues, and it was also used to test the differences of EI value for different time durations. Furthermore, the differences of the average percentages of water, fat and protein among these three tissues were also identified by using one-way ANOVA. All statistical analyses were performed by using statistics software (Stata version 11.0, Stata), and *P* < 0.05 was considered to be statistically significant different. Otherwise, *P* value less than 0.10 was considered as borderline significant [27].

## Results

### Tissue content

**Figure 2:**
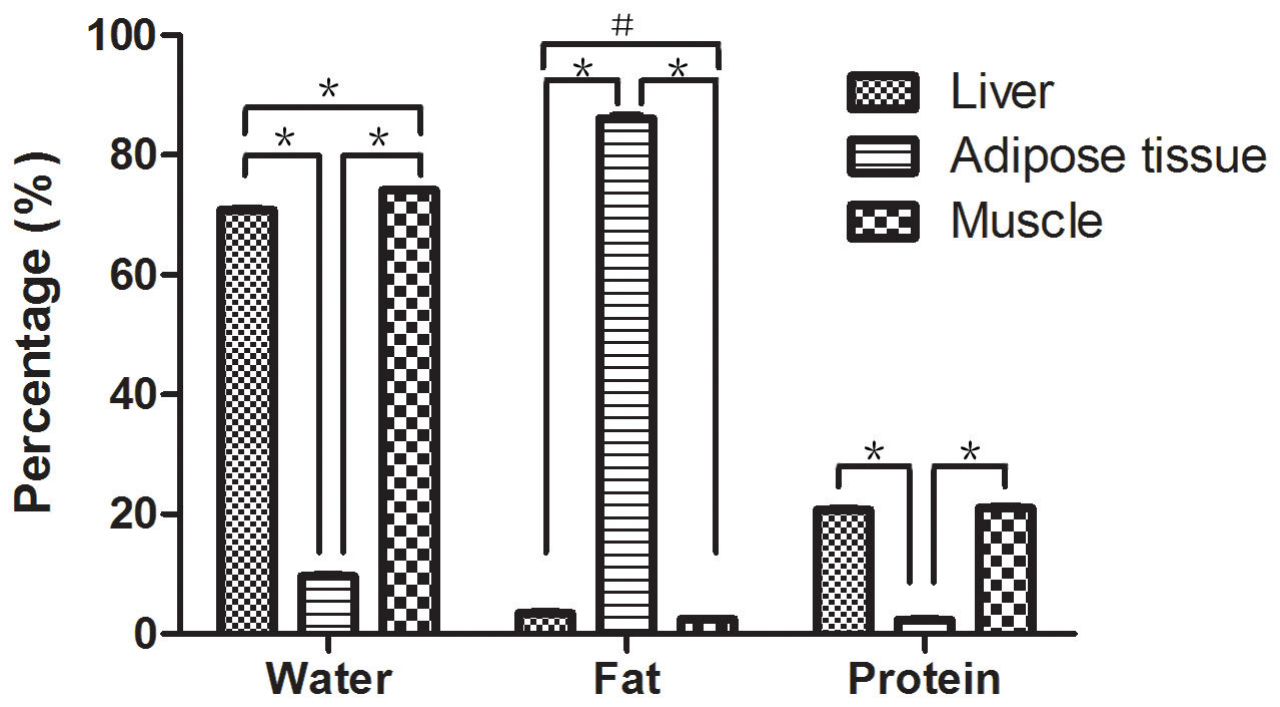
The average percentages of water, fat and protein in liver, muscle and adipose tissue (*P < 0.001; ＃*P* < 0.05).

The average percentages of water, fat and protein in muscle, liver and adipose tissue were tested, and they were significantly different (*P* < 0.001) in these three different tissues (Figure 2). The average percentages of water in muscle, liver and adipose tissue were 74.09% ± 0.13, 70.78% ± 0.24 and 9.62% ± 0.18, respectively, and they were significantly different from each other (*P* < 0.001). The average percentage of fat in adipose tissue (86.10% ± 0.47) was significantly higher than that in muscle (2.44% ± 0.09) and liver (3.48% ± 0.18) (*P* < 0.001). Furthermore, the average percentage of fat in liver was also higher than that in muscle (*P* = 0.014). The average percentages of protein in muscle (21.01% ± 0.25) and liver (20.73% ± 0.12) were significantly higher than that in adipose tissue (2.41% ± 0.07) (*P* < 0.001). However, the average percentages of protein in muscle and liver were not significantly different (*P* = 0.204).

### Size and shape of coagulation zone

**Figure 3:**
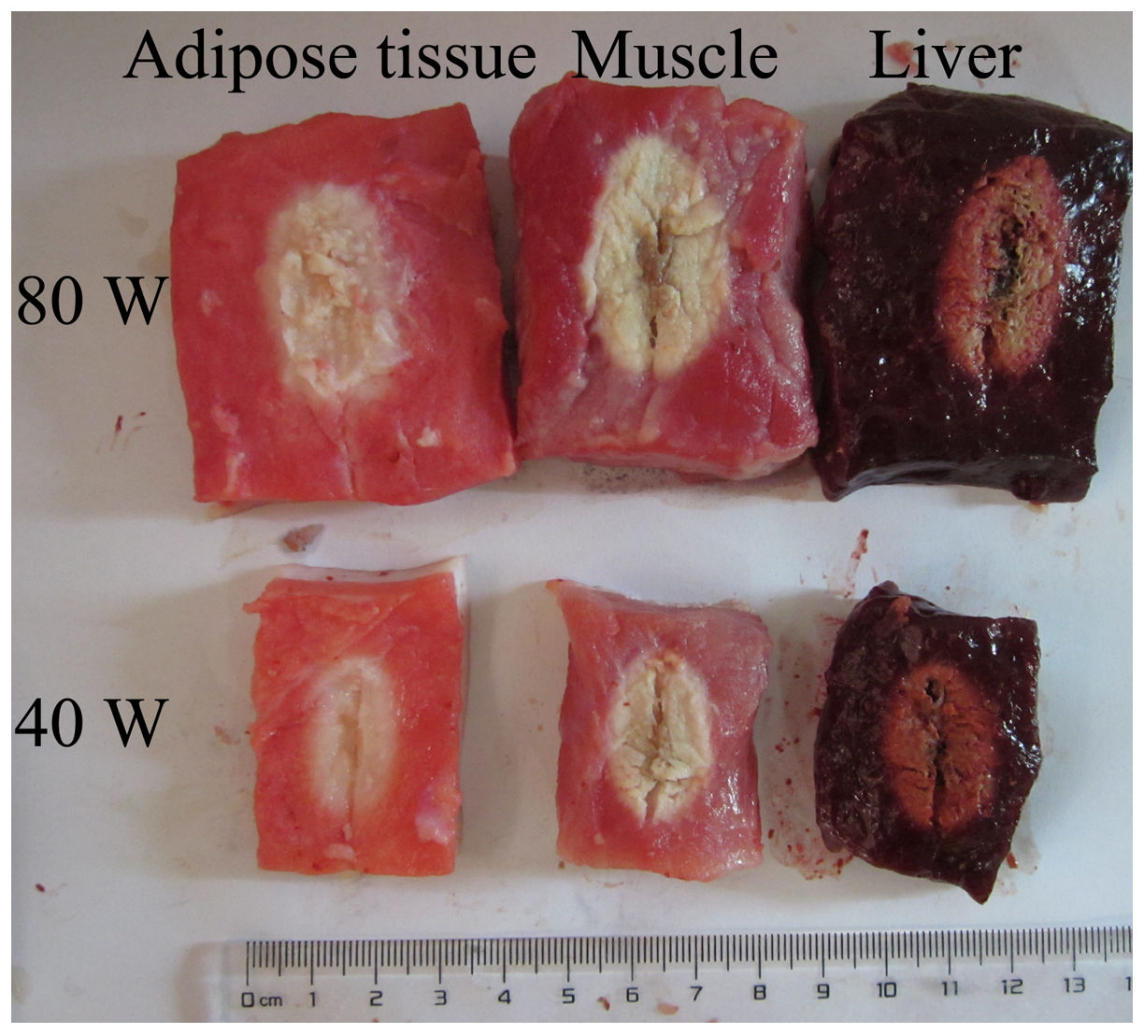
The coagulation zone (white zone) in adipose tissue, muscle and liver after 2,3,5-triphenyl tetrazolium chloride stain at two power output settings (40 W and 80 W) for 3 minutes of time duration.

**Figure 4:**
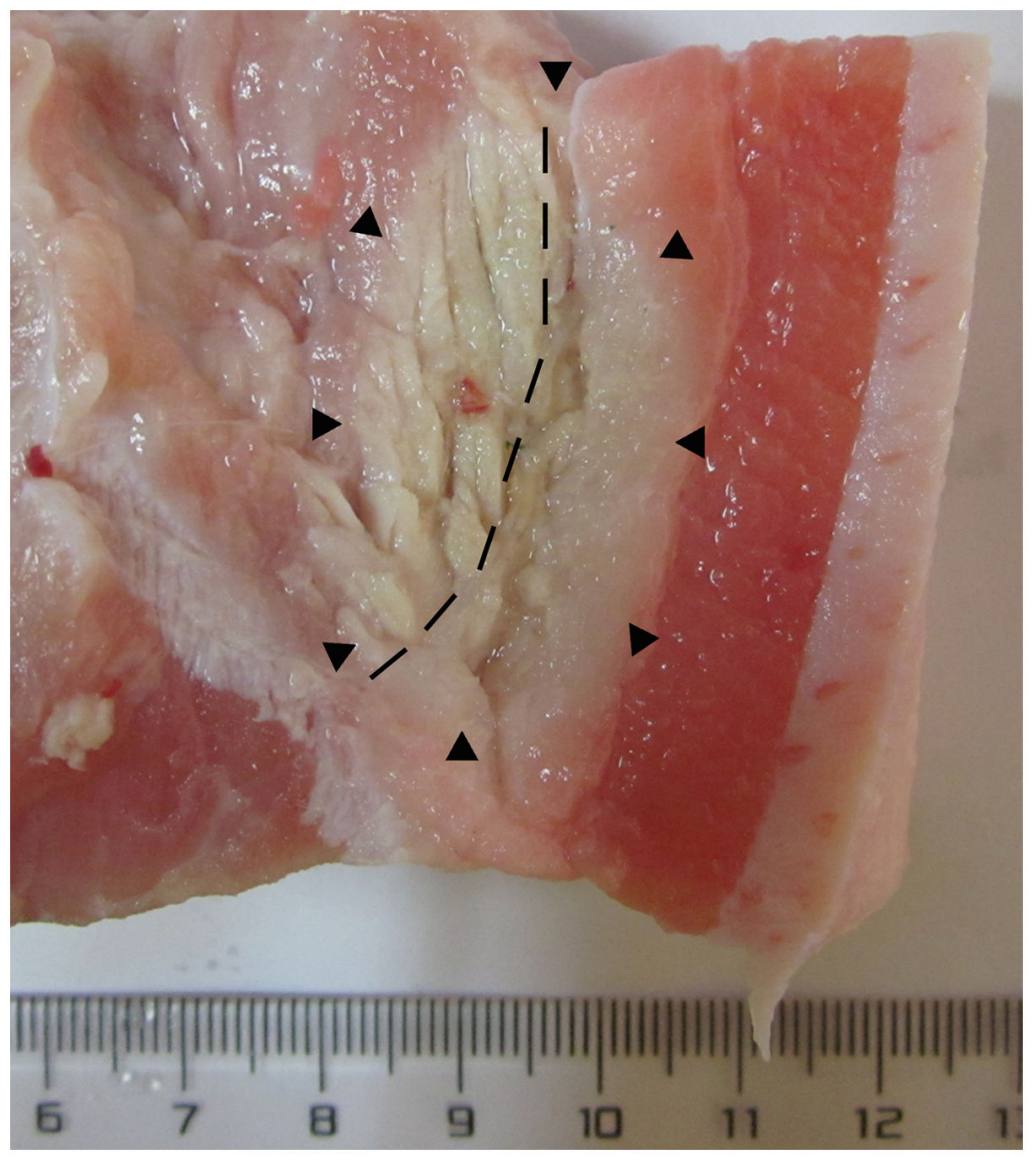
The coagulation zone (white zone) in the tissue containing both muscle (left) and adipose tissue (right) after 2,3,5-triphenyl tetrazolium chloride stain at 80 W for 3 minutes. The dashed line was the borderline between the muscle and adipose tissue.

The long-axis diameter and short-axis diameter of the three different tissues were compared at two power increments (40 and 80 W) and five time increments (1, 3, 5, 7, and 10 minutes). The detailed information about the two diameters was shown in Table 1. The long-axis diameters among the three tissues at each time-power combination were not significantly different (all *P* > 0.10). The short-axis diameters among the three tissues at 80 W for 1 minute (*P* = 0.0593) and 3 minutes (*P* = 0.0859) were borderline significantly different. Except for these two time-power combinations, the short-axis diameters among the three tissues at other time-power combinations were not significantly different (all *P* > 0.10). Figure 3 shows the coagulation zone in adipose tissue, muscle and liver after 2,3,5-triphenyl tetrazolium chloride stains at same time-power combinations. The coagulation zones were all elliptical in muscle, liver and adipose tissue. When microwave ablation was performed in the tissue containing both muscle and adipose tissue, the coagulation zone was also elliptical (Figure 4).

**Table 1:** Diameters of coagulation zones for microwave antennae at 40 W and 80 W.

**Power**	**Long axis** (mean ± SD, cm)				**Short axis** (mean ± SD, cm)			
	**Muscle**	**Liver**	**Adipose tissue**	***P* value**	**Muscle**	**Liver**	**Adipose tissue**	***P* value**
40 W								
1 min	2.27±0.15	2.4±0.10	2.13±0.21	0.2051	1.20±0.20	1.17±0.06	1.03±0.06	0.2963
3 min	2.87±0.15	2.77±0.12	2.87±0.23	0.7290	1.80±0.10	1.63±0.06	1.67±0.12	0.1517
5 min	3.20±0.10	3.07±0.06	3.13±0.21	0.5305	2.40±0.17	2.07±0.12	2.17±0.21	0.1226
7 min	3.37±0.06	3.50±0.10	3.50±0.10	0.1828	2.60±0.20	2.53±0.12	2.37±0.06	0.1820
10 min	3.63±0.12	3.57±0.06	3.80±0.17	0.1394	2.67±0.06	2.73±0.15	2.80±0.10	0.3944
80 W								
1 min	2.57±0.06	2.63±0.06	2.67±0.06	0.1780	1.60±0.10	1.43±0.15	1.30±0.10	0.0593
3 min	3.27±0.21	3.43±0.12	3.40±0.10	0.4064	2.23±0.06	2.30±0.10	2.13±0.06	0.0859
5 min	3.63±0.12	3.57±0.12	3.77±0.12	0.1780	2.63±0.25	2.60±0.10	2.37±0.06	0.1642
7 min	4.20±0.36	4.17±0.15	4.07±0.12	0.7793	2.83±0.12	2.87±0.06	2.67±0.12	0.1009
10 min	4.67±0.06	4.50±0.10	4.70±0.26	0.3542	3.23±0.15	3.20±0.10	3.13±0.32	0.8470

The shape of the coagulation zone is very important for complete ablation. The EI value was used to describe the shape of the coagulation zone in this study (Table 2). The EI value at 40 W for 1 minute of treatment duration was 2.01 ± 0.16, and it decreased to 1.34 ± 0.08 for 10 minutes of treatment duration (*P* < 0.05). The EI values at 40 W of 5, 7 and 10 minutes were not significantly different from each other (*P* > 0.05). At 80 W, the EI value for 1 minute of treatment duration was 1.84 ± 0.25, and it decreased to 1.45 ± 0.08 for 10 minutes of treatment duration (*P* < 0.05). Furthermore, the EI values of 3, 5, 7 and 10 minutes were not significantly different from each other (*P* > 0.05). Microwave ablations with ≥ 5 minutes time duration can induce coagulation zones with clinically desirable ablation shape at both 40 W and 80 W.

**Table 2:** Ellipticity index of microwave ablation zones.

**Variables**	**40 W** (mean ± SD)				**80 W** (mean ± SD)			
	**Muscle**	**Liver**	**Adipose tissue**	**Average**	**Muscle**	**Liver**	**Adipose tissue**	**Average**
1 min	1.91±0.19	2.06±0.05	2.07±0.21	2.01±0.16	1.61±0.13	1.85±0.23	2.06±0.17	1.84±0.25
3 min	1.59±0.10	1.69±0.01	1.72±0.13	1.67±0.10	1.46±0.06	1.49±0.03	1.60±0.09	1.52±0.08
5 min	1.34±0.14	1.49±0.07	1.46±0.22	1.43±0.15	1.39±0.14	1.37±0.07	1.59±0.04	1.45±0.13
7 min	1.30±0.08	1.38±0.04	1.48±0.02	1.39±0.09	1.49±0.18	1.45±0.03	1.53±0.02	1.49±0.10
10 min	1.36±0.06	1.31±0.07	1.36±0.11	1.34±0.08	1.45±0.08	1.41±0.07	1.50±0.07	1.45±0.08

### Histological findings

In gross specimens, the microwave ablated area appeared as an elliptical whitish zone surrounded by normal red muscle or liver. Similar to previous studies [20,21], three distinct zones were observed in each ablated area in muscle and liver, which included charring zone (the center zone), coagulation zone (white zone), and congestion zone (red zone). The charring zone increased when the treatment duration increased. However, the ablated area in the adipose tissue cannot be identified without 2,3,5-triphenyl tetrazolium chloride stain, and no charring was observed in adipose tissue in the present study. After 2,3,5-triphenyl tetrazolium chloride stain, the white ablated zone in adipose tissue can be identified clearly surrounded by red area in this study.

## Discussion

We firstly report results of microwave ablation in completely different tissues in *ex vivo* models. These results suggest that the long-axis and short-axis diameters of microwave coagulation zones were not significantly different from each other in three different tissues (muscle, liver and adipose tissue) in *ex vivo* porcine models. Furthermore, we also assessed the shape of the coagulation zone for different time durations. The present study shows that the EI value decreased when the time duration increased.

Tissue electrical and thermal conductivities are very important during RFA [16,28]. Liver is a homogenous organ; however, breast is more heterogeneous (dense pattern, adipose pattern or mixed pattern) [14,29]. Therefore, breast cancer RFA is a complex procedure. A previous study suggests microwave ablation zone was less affected by tissue heterogeneity than that in RFA [17]. Muscle, liver, and adipose tissue are completely different tissues with different contents. Muscle and liver are high-water high-ion-content tissues, while adipose tissue is low-water low-ion-content [15,29]. Our results indicate that the extent of microwave coagulation zone among muscle, liver and adipose tissue was the same. It is important to distinguish between direct microwave heating and conductive heating in creating ablation zone. Microwave ablation zone in this study was created by direct microwave heating. Microwave ablation may be not influenced by the content of the tissue and is suitable for breast tumor ablation regardless of components of the breast. Although our results were encouraging, future studies are still needed to determine whether microwave ablation zone is affected by the content of the tissues.

Furthermore, the extent of microwave coagulation zone might be associated with high blood flow *in vivo* [18]. Theoretically, a smaller ablation volume can be achieved in high blood-flow tissues relative to low blood flow tissues. Sommer CM and colleagues found that after interruption of blood flow, microwave ablation areas were significantly larger than those achieved without limitation of tissue perfusion [30]. Our ex vivo model was not influenced by blood-flow, and we can’t ablate completely different tissues in clinical practice. However, before applied in clinical practice, the association between blood flow and the extent of the ablated tissue must be closely monitored.

For complete ablation, the shape of the tumor and the microwave zone should be considered. The EI was used in the present study to describe the shape of the microwave coagulation zone. Similar to a previous *ex vivo* study [31], the EI value of 1 minute treatment duration was higher than that of 10 minutes treatment duration regardless of the output power. Furthermore, the EI value did not decrease significantly when the treatment duration was more than 5 minutes. Microwave ablations with more than 3 minutes time duration can induce coagulation zones with desirable shape for clinical practice. Consistent with previous studies [30,32], the shapes among different output powers were comparable. The characteristics of the coagulation zone should be considered when microwave ablation is applied in clinical practice. In clinical practice, the short-axis diameter of the tumor should not be ignored [33]. The coagulation zone and the treatment duration should be estimated before microwave ablation according to the shape, the size of the tumor, and the characteristics of microwave ablation.

Several limitations still exist in the present study. First, our study was conducted in *ex vivo* porcine models for relatively short time-durations, and our results should be confirmed in *in vivo* studies for a long time-duration. Second, the association between blood-flow and the extent of microwave coagulation zone was not assessed in this study. Third, the experimental model cannot mimic the different breast tissue perfectly. Forth, there was no temperature monitoring in this experiment. Fifth, although our results were encouraging, long-term effect of microwave ablations in different tissue was not assessed.

## Conclusions

In conclusion, the contents of water, fat and protein were significantly different in porcine muscle, liver and adipose tissue. Importantly, the extent of microwave ablation zones was not significantly different among completely different tissues at all time-power combinations. The EI value significantly decreased when the treatment duration increased from 1 minute to 10 minutes regardless of the output power. Microwave ablations with ≥ 5 minutes time duration can induce coagulation zones with clinically desirable ablation shape at both 40 W and 80 W. Although future clinical studies are still required to validate our encouraging results, microwave ablation may be a promising modality.
